# Markers of limbic system damage following SARS-CoV-2 infection

**DOI:** 10.1093/braincomms/fcad177

**Published:** 2023-06-13

**Authors:** Marine Thomasson, Philippe Voruz, Alexandre Cionca, Isabele Jacot de Alcântara, Anthony Nuber-Champier, Gilles Allali, Lamyae Benzakour, Patrice H Lalive, Karl-Olof Lövblad, Olivia Braillard, Mayssam Nehme, Matteo Coen, Jacques Serratrice, Jean-Luc Reny, Jérôme Pugin, Idris Guessous, Basile N Landis, Alessandra Griffa, Dimitri Van De Ville, Frederic Assal, Julie A Péron

**Affiliations:** Clinical and Experimental Neuropsychology Laboratory, Faculty of Psychology, University of Geneva, Geneva 1205, Switzerland; Neurology Department, Department of Clinical Neurosciences, Geneva University Hospitals, Geneva 1205, Switzerland; Clinical and Experimental Neuropsychology Laboratory, Faculty of Psychology, University of Geneva, Geneva 1205, Switzerland; Neurology Department, Department of Clinical Neurosciences, Geneva University Hospitals, Geneva 1205, Switzerland; Faculty of Medicine, University of Geneva, Geneva 1011, Switzerland; Clinical and Experimental Neuropsychology Laboratory, Faculty of Psychology, University of Geneva, Geneva 1205, Switzerland; Clinical and Experimental Neuropsychology Laboratory, Faculty of Psychology, University of Geneva, Geneva 1205, Switzerland; Neurology Department, Department of Clinical Neurosciences, Geneva University Hospitals, Geneva 1205, Switzerland; Clinical and Experimental Neuropsychology Laboratory, Faculty of Psychology, University of Geneva, Geneva 1205, Switzerland; Leenaards Memory Centre, Lausanne University Hospital and University of Lausanne, Lausanne 1205, Switzerland; Psychiatry Department, Geneva University Hospitals, Geneva 1205, Switzerland; Neurology Department, Department of Clinical Neurosciences, Geneva University Hospitals, Geneva 1205, Switzerland; Faculty of Medicine, University of Geneva, Geneva 1011, Switzerland; Faculty of Medicine, University of Geneva, Geneva 1011, Switzerland; Diagnostic and Interventional Neuroradiology Department, Geneva University Hospitals, Geneva 1205, Switzerland; Division and Department of Primary Care Medicine, Geneva University Hospitals, Geneva 1205, Switzerland; Division and Department of Primary Care Medicine, Geneva University Hospitals, Geneva 1205, Switzerland; Division of General Internal Medicine, Department of Medicine, Geneva University Hospitals and Geneva University, Geneva 1205, Switzerland; Division of General Internal Medicine, Department of Medicine, Geneva University Hospitals and Geneva University, Geneva 1205, Switzerland; Faculty of Medicine, University of Geneva, Geneva 1011, Switzerland; Division of General Internal Medicine, Department of Medicine, Geneva University Hospitals and Geneva University, Geneva 1205, Switzerland; Faculty of Medicine, University of Geneva, Geneva 1011, Switzerland; Intensive Care Department, Geneva University Hospitals, Geneva 1205, Switzerland; Faculty of Medicine, University of Geneva, Geneva 1011, Switzerland; Division and Department of Primary Care Medicine, Geneva University Hospitals, Geneva 1205, Switzerland; Faculty of Medicine, University of Geneva, Geneva 1011, Switzerland; Rhinology-Olfactology Unit, Otorhinolaryngology Department, Geneva University Hospitals, Geneva 1205, Switzerland; Leenaards Memory Centre, Lausanne University Hospital and University of Lausanne, Lausanne 1205, Switzerland; Institute of Bioengineering, Centre for Neuroprosthetics, Ecole Polytechnique Fédérale de Lausanne (EPFL), Lausanne 1015, Switzerland; Department of Radiology and Medical Informatics, University of Geneva, Geneva 1205, Switzerland; Faculty of Medicine, University of Geneva, Geneva 1011, Switzerland; Institute of Bioengineering, Centre for Neuroprosthetics, Ecole Polytechnique Fédérale de Lausanne (EPFL), Lausanne 1015, Switzerland; Neurology Department, Department of Clinical Neurosciences, Geneva University Hospitals, Geneva 1205, Switzerland; Faculty of Medicine, University of Geneva, Geneva 1011, Switzerland; Clinical and Experimental Neuropsychology Laboratory, Faculty of Psychology, University of Geneva, Geneva 1205, Switzerland; Neurology Department, Department of Clinical Neurosciences, Geneva University Hospitals, Geneva 1205, Switzerland

**Keywords:** post-COVID syndrome, emotion, neuropsychological deficits, MRI, functional connectivity

## Abstract

Alterations of the limbic system may be present in the chronic phase of SARS-CoV-2 infection. Our aim was to study the long-term impact of this disease on limbic system–related behaviour and its associated brain functional connectivity, according to the severity of respiratory symptoms in the acute phase. To this end, we investigated the multimodal emotion recognition abilities of 105 patients from the Geneva COVID-COG Cohort 223 days on average after SARS-CoV-2 infection (diagnosed between March 2020 and May 2021), dividing them into three groups (severe, moderate or mild) according to respiratory symptom severity in the acute phase. We used multiple regressions and partial least squares correlation analyses to investigate the relationships between emotion recognition, olfaction, cognition, neuropsychiatric symptoms and functional brain networks. Six to 9 months following SARS-CoV-2 infection, moderate patients exhibited poorer recognition abilities than mild patients for expressions of fear (*P* = 0.03 corrected), as did severe patients for disgust (*P* = 0.04 corrected) and irritation (*P* < 0.01 corrected). In the whole cohort, these performances were associated with decreased episodic memory and anosmia, but not with depressive symptoms, anxiety or post-traumatic stress disorder. Neuroimaging revealed a positive contribution of functional connectivity, notably between the cerebellum and the default mode, somatosensory motor and salience/ventral attention networks. These results highlight the long-term consequences of SARS-Cov-2 infection on the limbic system at both the behavioural and neuroimaging levels.

## Teaser

Thomasson *et al*. reported that patients performed poorly on multimodal emotion recognition 6–9 months after SARS-CoV-2 infection. This poor performance was associated with functional MRI markers, particularly in structures within the limbic and cerebellar systems. Taken together, the results may provide evidence of long-term damage by SARS-CoV-2 to the CNS.

## Graphical Abstract

**Graphical abstract:**
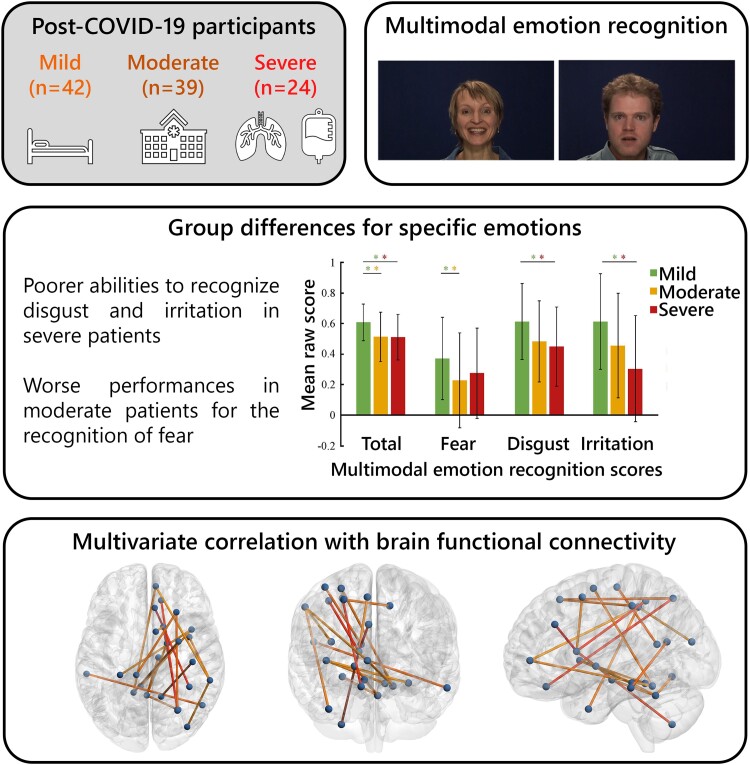

See A. Matias-Guiu and Díez-Cirarda (https://doi.org/10.1093/braincomms/fcad189) for a scientific commentary on this article.

## Introduction

Behavioural and neuroimaging data point to alterations of the limbic system in the chronic phase of COVID-19.^1–6^ Regarding commonly reported sequelae other than the well documented olfactory^7^ and episodic memory disorders,^1,2,8–12^ evidence has emerged suggesting diminished emotion recognition abilities.^1,13^ Authors have recently shown that some patients who had moderate or severe symptoms in the acute phase of COVID-19 exhibit a general reduction in their ability to recognize emotional stimuli 6–9 months later.^2,8^ This suggests an effect of SARS-CoV-2 infection on affective recognition abilities that goes beyond the impact of hospitalization in an intensive care unit (ICU),^14^ but to date, no study has sought to determine whether these results are driven by specific emotions. Neuroimaging studies in patients with COVID-19 have used a variety of methods, including fluorodeoxyglucose (^18^FDG) PET and MRI, to highlight hypometabolism or hypoconnectivity in cortical–subcortical brain regions and networks,^1,3–5,13,15^ many belonging to the limbic system, as well as alterations in the cerebellum.^16^ Moreover, a recent study highlighted a reduction in grey-matter thickness in the orbitofrontal cortex and parahippocampal gyrus, as well as tissue damage in regions that are functionally connected to the primary olfactory cortex—regions that are also known to form part of the limbic system.^17^ However, only a small number of studies have correlated cognitive and neuropsychiatric variables with functional neuroimaging data,^18^ and studies of emotion recognition abilities have so far focused solely on behavioural data.^2,8^ None have explored the relationships between multimodal emotion recognition and structural and functional neuroimaging data.

Although previous results suggest that the virus directly (or more likely indirectly) attacks the CNS, and presumably the limbic system in particular, the potential effect of neuropsychiatric symptoms on long-term limbic-related functions has yet to be investigated. Many neuropsychiatric disorders, including post-traumatic stress disorder (PTSD), anxiety and depressive symptoms, have been described following SARS-CoV-2 infection, as well as fatigue and sleep disturbances,^19^ and are well known to have an impact on emotion recognition abilities.^20–24^ To the best of our knowledge, no study has yet assessed the impact of relevant secondary neuropsychiatric variables on the recognition of individual multimodal emotions, as a function of the severity of respiratory symptoms in the acute phase. In this context, tasks assessing emotion processing abilities could be of interest. Only two studies have behaviourally assessed long-term emotion recognition abilities following SARS-CoV-2 infection, highlighting reduced performance in hospitalized versus non-hospitalized groups of patients.^2,8^ That said, these studies were carried out on overall scores and did not assess performances for individual emotions.

According to recent models of emotions, particularly the process model of emotions, each emotion has distinct properties and involves five distinct functions (appraisal, automatic physiology, action tendencies, motor expression and subjective feeling).^25^ The limbic circuits, involving the orbitofrontal cortex, insula and amygdala, inferior temporal lobe and subcortical regions, have historically been described as forming the neural base of emotional processes, as well as memory functions.^26,27^ Nevertheless, recent evidence suggests that there is not one limbic system, but several differentiated limbic systems for emotional processes, involving anterior structures of the limbic system, orbitofrontal cortex and amygdala in emotion processing, reward assessment and decision-making.^28^ Meanwhile, memory functions are underpinned by the hippocampus and the limbic structures to which it is connected, including the posterior cingulate cortex and the fornix–mammillary body–anterior thalamus–posterior cingulate circuit.^28^ Finally, these processes also seem to be underpinned by cerebellar structures, highlighting the close interaction between the limbic systems and the cerebellum.^29^

In this context, the present study was conducted to examine the impact of the severity of respiratory symptoms in the acute phase of COVID-19 on emotion recognition abilities 6–9 months post-infection (aim 1) and to explore the influence of secondary behavioural variables (e.g. olfaction, memory or depressive symptoms) (aim 2), as well as functional brain connectivity (aim 3). To this end, we assessed multimodal emotion recognition in 105 patients 223.07 ± 41.69 days following SARS-CoV-2 infection. We ran regression analyses to investigate the potential predictive value of neuropsychological functions sustained by the limbic system (verbal and visual episodic memory), neuropsychiatric manifestations (PTSD, anxiety, depressive symptoms, fatigue and sleep disorders) and olfactory performances. Finally, 45 of these 105 patients underwent functional MRI, and exploratory partial least squares correlation (PLSC) analyses were performed to identify associations between multimodal emotion recognition abilities and functional brain networks.

## Materials and methods

### Participants

Data from 105 patients were extracted from the COVID-COG database of Geneva University Hospitals (HUG).^1,2,8,13,30^ For each patient, we carried out a medical file review, followed by a telephone call inviting the patient to take part in the study, if all the eligibility criteria were met. Exclusion criteria were a history of neurological issues, neuropsychiatric disorders, cancer, neurodevelopmental pathologies, pregnancy and age above 80 years.

The patients had all been diagnosed with SARS-CoV-2 infection between March 2020 and May 2021, either by positive PCR from a nasopharyngeal swab and/or by positive serology. Patients were included in the study 223.07 ± 41.69 days post-infection and divided into the following 3 groups: 24 patients who had been in ICU during the acute phase of the infection (severe), 39 patients who had been hospitalized but did not require mechanical ventilation (moderate) and 42 patients who had tested positive but had not been hospitalized (mild). During the screening–inclusion process, because of the limited number of patients who had been in ICU and who met our inclusion criteria, the mild and moderate groups were matched with the severe group for age, sociocultural level, gender and clinical variables (except for sleep apnoea and chronic kidney disease). All descriptive data are provided in Table 1.

**Table 1:** Sociodemographic data and medical history

	Mild *n* = 44	Moderate *n* = 42	Severe *n* = 24	*P*-value
Mean age in years (± *SD*)	55.45 (± 8.76)	56.13 (± 10.30)	61.48 (± 11.92)	0.110
Mean education level (1–3) (± *SD*)	2.77 (± 0.42)	2.63 (± 0.59)	2.52 (± 0.59)	0.192
Gender (% men)	65.91	63.15	73.91	0.420
Handedness (% right handed)	97.70	92.90	95.80	0.553
Mean days of hospitalization (± *SD*)	–	11.38 (± 12.60)	40.52 (± 32.72)	–
Diabetes (%)	2.30	10.50	21.70	0.075
Smoking (%)	13.60	2.60	4.30	0.138
History of respiratory disorders (%)	13.50	10.50	26.10	0.242
History of cardiovascular disorders (%)	13.60	15.80	21.70	0.691
History of neurological disorders (%)	0	0	0	1
History of psychiatric disorders (%)	4.5^a^	0^a^	4.30^a^	0.416
History of cancer (%)	0	0	0	1
History of severe immunosuppression (%)	0	0	0	1
History of developmental disorders (%)	0	0	0	1
Chronic kidney disease (%)	0	0	8.3	0.026*
Sleep apnoea syndrome (%)	9.10	10.50	30.40	0.043*

Statistical analysis performed: Kruskal–Wallis or chi^2^.

SD, standard deviation.

a Treated depression more than 10 years prior to COVID-19.

### Standard protocol approvals, registrations and patient consents

All participants gave their written informed consent, and the study was approved by the cantonal ethics committee of Geneva (CCER-02186).

### Measures

For the purpose of the present study, we extracted olfactory, multimodal emotion recognition, memory and neuropsychiatric data,^1,8,13,30^ as well as neuroimaging data^1,13^ from the COVID-COG project database.^9^

### Multimodal emotion recognition

**Figure 1:**
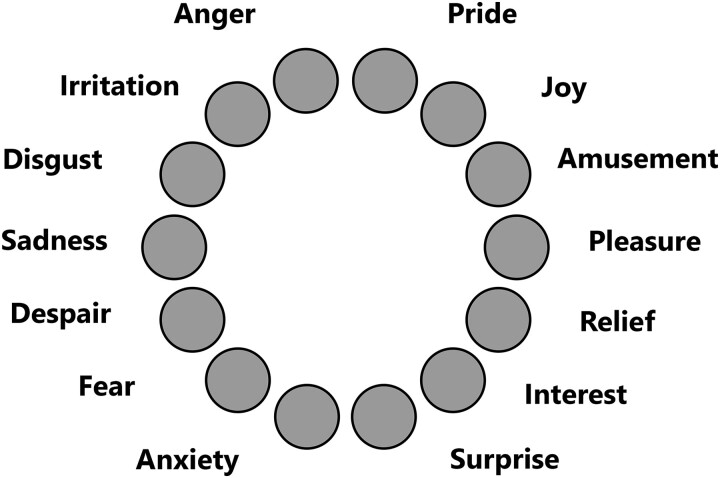
**Response format of the short form of the Geneva Emotion Recognition Test.** After watching each video clip, the participant must choose the answer from the choices provided in this wheel.

We used the short form of the Geneva Emotion Recognition Test (GERT-S^31^), a validated multimodal emotion recognition task adapted from the full 83-item GERT. GERT-S is a performance-based test that measures individual differences in people’s ability to recognize others’ emotions expressed in the face, voice or body. During this task, participants watched 42 short video clips with sound (duration: 1–3 s), in which 14 different emotions extracted from the validated GEMEP database^32^ were displayed (see Fig. 1). Of the 14 emotions, 12 could be contrasted with respect to valence and arousal: emotions of joy, amusement, pride (high arousal/positive valence), pleasure, relief, interest (low arousal/positive valence), anger, fear, despair (high arousal/negative valence) and irritation, anxiety and sadness (low arousal/negative valence). The remaining two emotions were disgust and surprise. Each emotion was expressed in three different video clips, in which actors expressed the emotion through facial, gestural or vocal cues. The vocal stimuli were meaningless speech (pseudowords), to avoid semantic aspects. Responses were scored as correct (1) or incorrect (0), such that the total possible GERT-S score ranged from 0 to 42. For the purpose of the study, multimodal emotion recognition data that had not previously been analysed (except for the total score) were extracted from the COVID-COG database.

### Secondary variables

#### Episodic memory

Verbal episodic memory was assessed with Grober and Buschke’s French-language 16-item Free and Delayed Recall Test (RL/RI-16).^33^ Based on short-term and long-term recall, this test allows users to distinguish between the different memory processes of encoding, retrieval and storage. Visual episodic memory was assessed with the delayed recall of the Rey–Osterrieth Complex Figure test.^34^ This test is divided into two phases: (i) a copy phase, which measures visuoconstructive function, and (ii) a recall phase, performed 3 and 20 min later (i.e. delayed recall 3′′ and delayed recall 20′′), which measures the extent and quality of recall of the original figure from short- or long-term spatial memory. If patients failed to produce a copy of Rey’s figure, we did not administer the delayed recall,^35^ as the assessment of visual memory would otherwise have been biased by visuoconstructive deficits. For this reason, 11 patients did not perform the memory recall, but all patients were included in the other analyses.

#### Olfaction

We administered the validated Sniffin’ Sticks test battery, where participants must identify 16 odours. For each odour, they can choose between four possible answers.^36^

#### Neuropsychiatric measures

All neuropsychiatric data were collected online (Qualtrics, 2010) in the week preceding the neuropsychological assessment, via standardized and computerized questionnaires specially designed for the study. More specifically, depressive symptoms were assessed with the Beck Depression Inventory-Second edition,^37^ anxiety with the State-Trait Anxiety Inventory,^37^ apathy and its distinct subtypes with the Apathy Motivation Index,^38^ PTSD with the Posttraumatic Stress Disorder Checklist for DSM-5,^39^ manic symptoms with the Goldberg Mania Inventory,^40^ dissociative symptoms in the patient’s daily life with the Dissociative Experience Scale,^41^ current stress perception with the Perceived Stress Scale-14 items,^42^ emotion regulation with the Emotion Regulation Questionnaire^43^ and susceptibility to others’ emotions with the Emotion Contagion Scale.^44^

#### Fatigue and sleep disorders

Finally, fatigue was assessed with the French version of the Fatigue Impact Scale,^45^ potential sleeping disorders with the Insomnia Severity Index^46^ and symptoms of sleepiness in daily life with the Epworth Sleepiness Scale.^47^

### Neuroimaging data

Anatomical T1-weighted magnetization-prepared rapid acquisition gradient echo (T1w MPRAGE) and resting-state functional (rs-fMRI) MRI data were extracted from the COVID-COG dataset for 45 participants (mild, *n* = 17; moderate, *n* = 19; severe, *n* = 9). Within this subset, no significant differences were found between the groups on either sociocultural level (mild, 2.71 ± 0.47; moderate, 2.76 ± 0.44; severe, 2.72 ± 0.47; *P* = 0.93), handedness (one left-handed participant in both the mild and severe groups) or age (mild, 54.47 ± 9.17 years; moderate, 57.06 ± 11.60 years; severe, 58.00 ± 11.87 years; *P* = 0.75), whereas gender ratios differed significantly, as there were no women in the severe group (mild, 41.18% women; moderate, 35.29% women; severe, 0% women; *P* = 0.04). In addition, no differences were observed on the intervals between infection and MRI (mild, 253.81 ± 44.37 days; moderate, 276.88 ± 51.94 days; severe, 284.00 ± 50.46 days; *P* = 0.37) and between neuropsychological testing and MRI (mild, 33.06 ± 22.17 days; moderate, 35.71 ± 25.14 days; severe, 52.27 ± 27.42 days; *P* = 0.15).

The image acquisition procedure is described elsewhere^1^ (details and parameters listed in Supplementary Information 1, Supplementary Tables 1 and 2). Preprocessing was performed using fMRIPrep 20.2.3,^48^ which is based on Nipype 1.6.1.^49^ In addition, the fMRI timeseries were detrended, low-frequency discrete cosine-basis regressors (≤0.005 Hz) were removed from the signal, a low-pass filter with a cut-off frequency of 0.15 Hz was applied, each fMRI volume was spatially smoothed with a Gaussian kernel (full width at half maximum = 4 mm), and frames with framewise displacement >0.7 mm were excluded.

Resting-state functional connectivity was computed as the Fisher-transformed Pearson’s correlation coefficient between the mean fMRI timecourses within 156 regions of interest associated with 19 resting-state networks^50^ (100 cortical,^51^ 34 cerebellar^52^ and 22 subcortical regions^53^).

### Statistical analysis

First, we performed intergroup comparisons between the severe, moderate and mild groups on the GERT-S total score and subscores, using nonparametric Kruskal–Wallis analyses of variance and (if significant) Mann–Whitney U-tests. Bilateral *P*-values were corrected using the Bonferroni criterion. In addition, to establish baseline emotion recognition performances within our cohort, we compared the performances of our three groups of patients with that of healthy control participants from a previous study where the GERT-S was administered to a normative population (German-speaking part of Switzerland) in March 2020 (during lockdown).^54^ The raw scores were converted to standardized scores (*z* scores, with a pathological cut-off score of −1.6), in accordance with the guidelines of the Swiss Association of Neuropsychology.^55,56^

Second, we performed forward stepwise multiple regressions on the significant emotion scores, adding verbal and visual episodic memory, neuropsychiatric, fatigue and olfactory scores as predictors. All the statistical analyses were run on TIBCO Statistica™ 14.0.0.

Third, to find associations between neuroimaging and behavioural data, we extracted multivariate components using a data-driven approach, performing exploratory group PLSC analysis on the whole cohort of patients.^57–60^ The resulting latent components were defined as linear combinations of whole-brain functional connectivity patterns and GERT performances (total score, fear, disgust and irritation emotion scores) with maximum covariances across patients and within each group. Additionally, the procedure was repeated separately for each group, to estimate the coherence of the group PLSC analysis. The significance of the multivariate correlations was assessed with permutation testing (1000 permutations) and the reliability of the features’ contributions (i.e. loadings) to the correlation components with bootstrapping (500 resamples). To control for the effect of memory processes on emotion recognition, we included the RL/RI 16-delayed free recall as a measure of verbal episodic memory, along with age, gender and sociocultural level as covariates. PLSC analyses were performed using the myPLS toolbox (https://github.com/danizoeller/myPLS).

To probe for structural alterations that might be associated with the functional patterns we observed, we ran voxel-based morphometry analyses, by computing the proportions of white- and grey-matter voxels in the whole brain or within parcels of our custom fMRI atlas. We used analyses of covariance (ANCOVA) to assess statistical differences between the groups, with age, gender and sociocultural level as covariates.

### Data availability

Nonsensitive COVID-COG data are available in open access on a dedicated platform (https://yareta.unige.ch/home, dataset DOI: 10.26037/yareta:56vcowyr7fdgxfikm5wycsc47a). The code for the PLSC analyses and figure generation is available in a GitHub repository (https://github.com/Cionkito/PLS-COVID_emotions).

## Results

### Multimodal emotion recognition 6-9 months post-infection according to symptom severity in the acute phase

**Figure 2:**
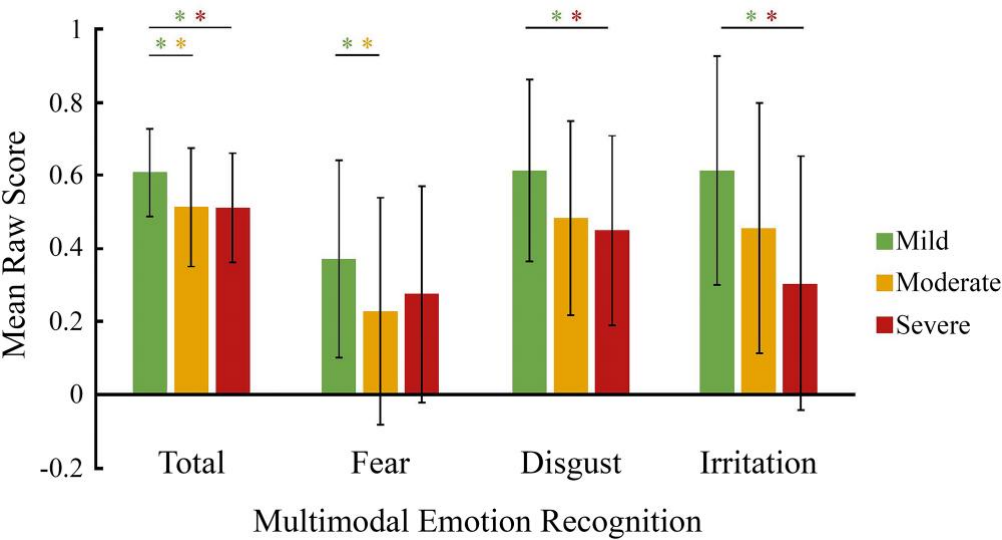
**Multimodal emotion recognition (as measured by the Geneva Emotion Recognition Test—short version) as a function of the severity of respiratory symptoms in the acute phase.** Bars represent raw mean scores for each emotion, and whiskers represent standard deviations. The results showed patterns of poorer multimodal recognition abilities in patients with a moderate or severe form in the acute phase as compared with patients who had a mild form in the acute phase. *Significant Mann–Whitney U-test differences between groups corrected for multiple comparisons (Bonferroni methods).

Results revealed a significant difference between the three groups (mild, moderate and severe) on the total emotion recognition score 6–9 months post-infection (*H* = 10.9, *P* < 0.01) (Fig. 2 and Supplementary Table 3). More specifically, mild patients performed better than both moderate (Cohen’s *d* = 3.33, η^2^ = 0.74, *z* = 2.9, *P* = 0.01) and severe (Cohen’s *d* = 2.89, η^2^ = 0.68, *z* = 2.6, *P* = 0.03) patients. This effect on the total score was mainly driven by performances for expressions of fear, irritation and disgust: mild patients performed better than moderate patients on fear expression recognition (Cohen’s *d* = 3.33, η^2^ = 0.74, *z* = 2.6, *P* = 0.03) and also performed better than severe patients on the recognition of expressions of irritation (Cohen’s *d* = 2.88, η^2^ = 0.68, *z* = 3.4, *P* < 0.01) and disgust (Cohen’s *d* = 0.67, η^2^ = 0.68, *z* = 2.5, *P* = 0.04).

When we compared the performances of our three patient groups with the performance of healthy control participants, we observed deficits for mild (9.52%), moderate (30.71%) and severe patients (45.83%) (see Supplementary Fig. 1 for more details).

### Clinical predictors of multimodal emotion recognition 6–9 months post-infection

**Figure 3:**
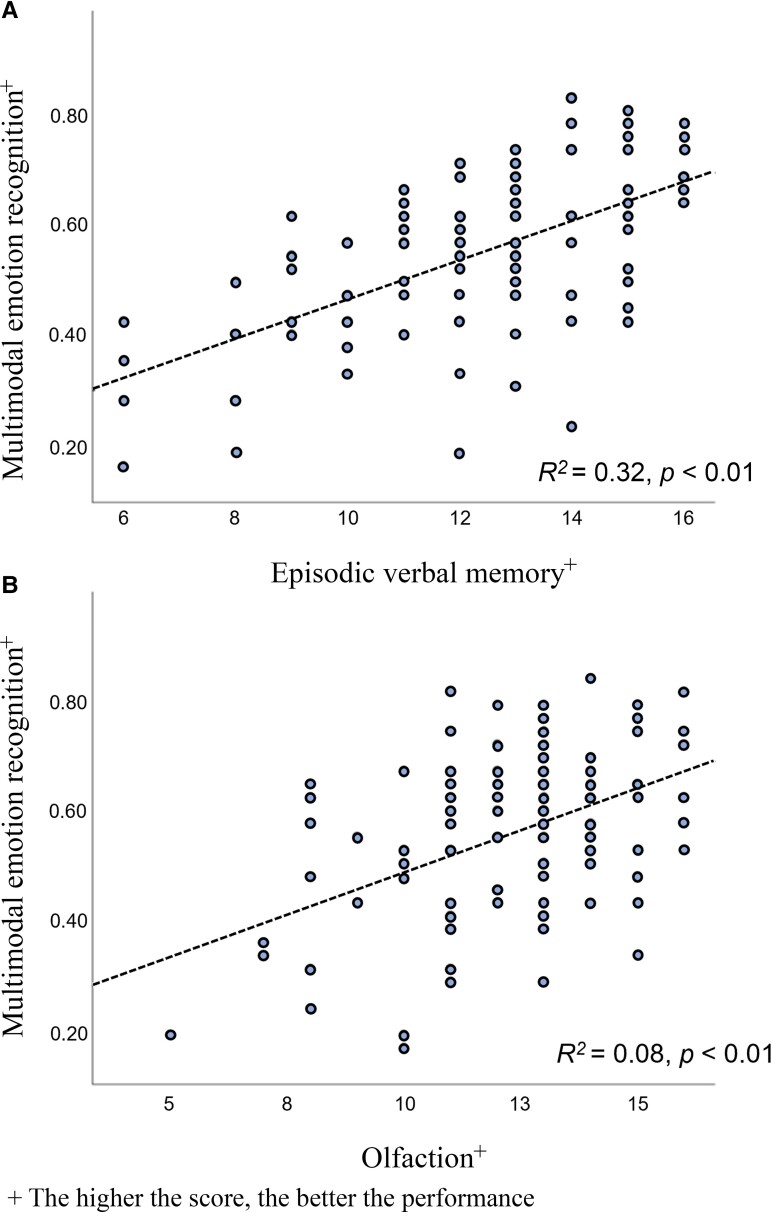
**Relationships between multimodal emotion recognition abilities (as measured by the total score of the Geneva Emotion Recognition Test—short version) and verbal episodic memory as measured by RL/RI 16-delayed free recall (top) and with olfaction as measured by the Sniffin’ Sticks test battery (*bottom*).** Each dot represents a patient; lines represent the best least square linear fits. (**A**) The poorer the ability to recognize emotions, the poorer the performance on verbal episodic memory task. (**B**) The poorer the ability to recognize emotions, the poorer the performance on the olfactory recognition test.

The variance inflation factor, which measures the correlations and the strength of these correlations, between predictor variables in a regression model, was calculated for each clinical predictor (Fig. 3). Analyses revealed values ranging from 1.00 to 2.72, indicating an acceptable level of multicollinearity in our regression model.^61,62^

For the GERT total score, the best fit was achieved using the following variables: RL/RI 16-delayed free recall (*R*^2^ = 0.32, *P* < 0.01), sniff test (anosmia) (*R*^2^ = 0.08, *P* < 0.01), Rey figure-delayed recall 3′′ (*R*^2^ = 0.06, *P* < 0.01), RL/RI 16-sum of three free recalls (*R*^2^ = 0.04, *P* < 0.01) and RL/RI 16-immediate recall (*R*^2^ = 0.02, *P* = 0.04).

For the GERT fear score, the best fit was achieved using the Rey figure-delayed recall 3′′ (*R*^2^ = 0.13, *P* < 0.01) and RL/RI 16-delayed free recall (*R*^2^ = 0.06, *P* = 0.01).

For the GERT disgust score, the best fit was achieved using the RL/RI 16-delayed total recall (*R*^2^ = 0.15, *P* < 0.01), RL/RI 16-delayed free recall (*R*^2^ = 0.05, *P* = 0.02) and Fatigue Impact Scale (physical fatigue) (*R*^2^ = 0.04, *P* = 0.04).

For the GERT irritation score, the best fit was achieved using the RL/RI 16-delayed free recall (*R*^2^ = 0.17, *P* < 0.01), sniff test (anosmia) (*R*^2^ = 0.04, *P* = 0.02) and RL/RI 16-sum of three total recalls (*R*^2^ = 0.04, *P* = 0.03).

### Functional and structural brain networks associated with emotion recognition performances

**Figure 4:**
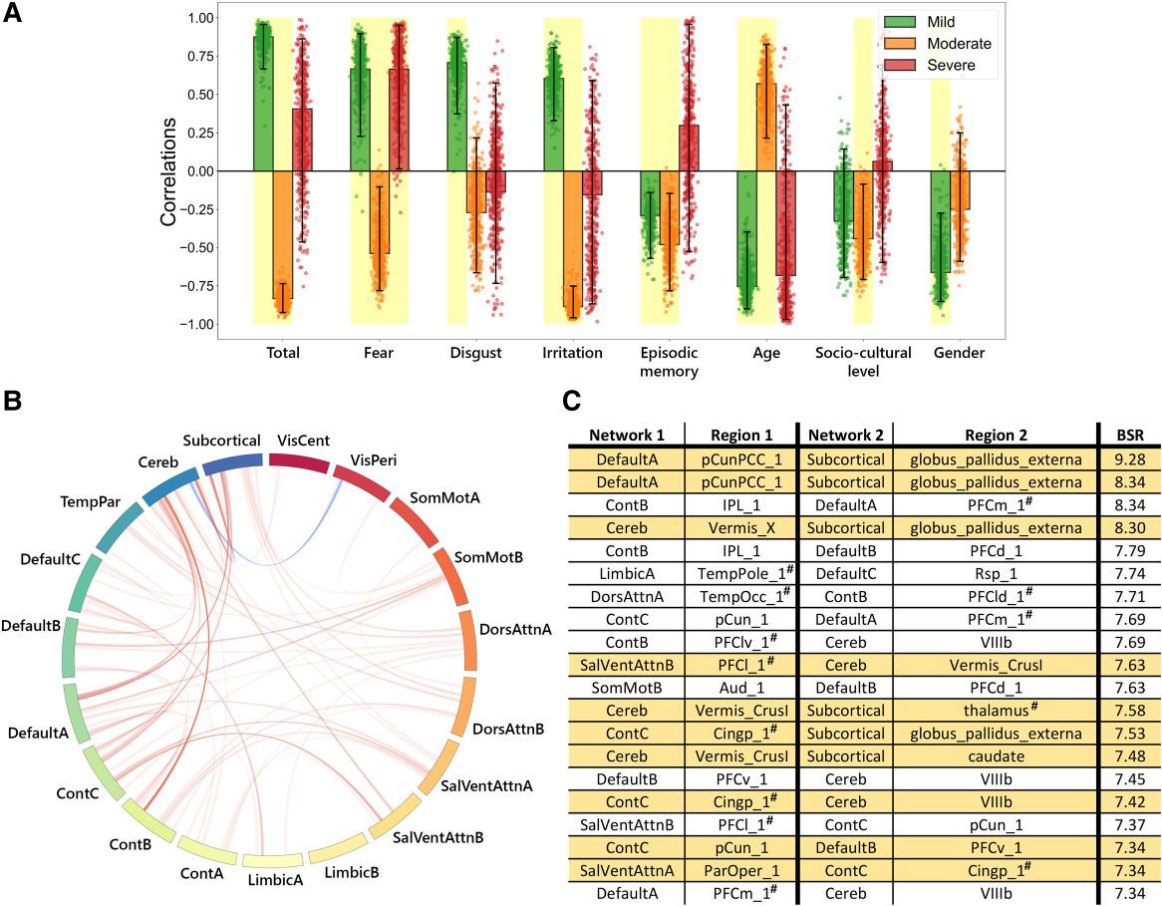
**Multivariate latent component from the group PLSC analysis.** (**A**) Loadings of behavioural data. PLSC loadings were defined as correlation coefficients between a given feature and its weight in the latent component. Dots represent samples from the bootstrap procedure, and yellow highlights indicate the reliability of the scores’ contributions to the multivariate correlation components. (**B**) Bootstrap sampling ratios for functional connectivity (5% highest positive and negative values evaluated with bootstrap). This network representation illustrates the neuroimaging pattern, with red links indicating a positive influence of functional connectivity on the latent component and darker colours indicating a higher number of significant connections for each resting-state networks’ pair involved in the pattern. (**C**) Twenty brain networks and regions whose connections had the greatest impact [bootstrap sampling ratio (BSR)] on the latent component. Regions: Aud, auditory cortex; IPL, inferior parietal lobule; PCC, post cingulate cortex; pCun, precuneus; PFC, prefrontal cortex; RSP, retrosplenial cortex; TempOcc, temporal occipital area.

The group PLSC analyses identified one significant component that survived false discovery rate correction (*P* = 0.004) (Fig. 4). This explained 35.63% of the covariance between emotion recognition scores and functional connectivity (Fig. 4A). The neuroimaging component revealed positive contributions of functional connections between the cerebellum and subcortical and cortical networks such as the default mode, somatosensory motor and salience/ventral attention networks (Fig. 4B and C). The same functional connectivity pattern was associated with two different behaviours in the patient group: higher total, fear, disgust and irritation recognition scores for mild patients and lower total, fear and irritation recognition scores for moderate patients. Moreover, the multivariate correlation was associated with younger age and female gender for mild participants and older age as well as lower sociocultural level for moderate patients. Finally, poorer verbal episodic memory, as measured by delayed total recall, remained stable for the mild and moderate groups. Results were comparable when verbal episodic memory was not included in the model (Supplementary Fig. 2). Separate PLSC analyses were performed for each group. They only yielded significant results for moderate patients (*P* = 0.021), and these did not survive false discovery rate correction (Supplementary Fig. 3). Even so, the multivariate pattern specific to moderate participants was congruent with the PLSC results (Supplementary Fig. 3). Finally, no structural differences were observed in the anatomical images that could be associated with the functional patterns (Supplementary Table 4).

## Discussion

The aim of the present study was 3-fold: *first*, to explore the impact of the severity of respiratory symptoms in the acute phase of COVID-19 on multimodal emotion recognition performances 6–9 months later; *second*, to determine whether memory, neuropsychiatric or olfactory variables are associated with these performances; and *third*, to identify the functional brain networks that are associated with them.

With respect to the first aim, we observed that both moderate and severe patients had significantly lower emotion recognition scores than mild patients. This result on the total score is consistent with previous results obtained with a smaller cohort^7^ and in a previous study performed on the COVID-COG cohort.^2^ However, for the first time to our knowledge, our results revealed poorer scores not just across the board, but for specific emotions (fear, irritation and disgust). These results support the hypothesis that the long-term neuropsychological effects of COVID-19 go beyond the well-known impact of hospitalization in ICU on cognitive functions, as attested by the performances displayed by the moderate group. However, this does not exclude an effect of conventional hospitalization.^1,8,9^ Thus, the ability to recognize emotions may be impaired following SARS-CoV-2 infection. Interestingly, using a transconnectome-based network diffusion model, Parsons *et al.*^63^ reported changes in thalamocortical connectivity in patients with COVID-19 that may have disrupted their regulation of consciousness and arousal, possibly affecting or interacting with emotion recognition.^64^

Regarding the second aim, multiple regression analyses revealed that the best predictors of multimodal emotion recognition were performances on verbal and visual episodic memory, as well as olfactory recognition abilities and self-reported physical fatigue. Surprisingly, neuropsychiatric symptoms, such as anxiety, depressive symptoms and PTSD, were not associated with emotion recognition abilities, despite showing high heterogeneity. Olfactory deficits (and potential damage to the olfactory system),^7^ as well as short- and long-term memory impairment post-infection,^1,8,11–13^ have previously been described following SARS-CoV-2 infection,^1,3,4,13,17^ but we are the first to demonstrate associations between these disorders and emotion recognition. Given the overlapping brain substrates underlying memory, olfactory and emotion recognition mechanisms,^65^ notably via the insula, amygdala and hippocampus,^66,67^ our observation of these associations is particularly relevant and is probably consistent with the idea of limbic system alterations following SARS-CoV-2 infection. Interestingly, this hypothesis is partially corroborated by the neuroimaging findings of the present study and of previous studies in other neuroimaging modalities (e.g. FDG-PET).

Regarding the third aim, we found significant correlations between multimodal emotion recognition performances and functional connectivity patterns involving cortico–subcortical–cerebellar networks overlapping the so-called limbic system. Although we identified a positive correlation between emotion recognition scores and functional connectivity in the mild group, there was a negative correlation in the moderate group and no correlation in the severe group. Our interpretation is that moderate to severe symptoms in the acute phase may induce a compensatory response in the limbic system and lead to the patterns of altered functional connectivity that can be observed at 6–9 months post-infection. Whatever the underlying processes, which remain to be explored in future studies, these results are consistent with our behavioural results, as well as with previous observations in the literature for the long-term consequences of SARS-CoV-2 infection using both fMRI^1,5,13^ and PET,^3,4,68^ and underline the involvement of the limbic system (probably the emotional and memory pathways) following SARS-CoV-2 infection.^1^ Interestingly, limbic system alteration following viral infections has already been reported, with an increased number of enzymes involved in inflammatory responses within limbic system and associated brain structures, such as the hippocampus and basal ganglia.^69,70^

### Limitations

It is important to note that the present study had five limitations. First, the smaller number of patients in the severe group who agreed to undergo an MRI (*n* = 9 patients) may explain the absence of significant correlations in the PLSC analyses. Nevertheless, the functional connectivity data of the severe patients were not particularly heterogeneous, compared with those of the mild and moderate groups (Supplementary Table 2). Further studies are needed to confirm the neuroimaging findings with a larger sample size. Second, by enrolling volunteers, we may have selected the most severe cases, even though a significant proportion of our sample did not report any complaints, as confirmed by the very low mean scores on the self-report cognitive complaint questionnaires, as previously observed in this cohort of patients.^1,2^ Third, between March 2020 and November 2020, the criteria for hospitalization may have changed, and some patients may therefore have been hospitalized for non-respiratory problems associated with COVID-19. That said, patient management was generally comparable, and all the patients included had the initial SARS-CoV-2 strain, as the variants only emerged later. Fourth, we did not include a control group, as the aim of the present study was to investigate differences in multimodal emotion recognition abilities and brain connectivity according to the severity of the acute infection. Therefore, we cannot exclude the possibility that the mild group would have exhibited reduced scores in comparison with a control group, as has been described in the literature. Finally, our moderate and severe groups may not have been representative of the population of hospitalized SARS-CoV-2 patients, owing to their lack of comorbidities (an inclusion criterion).

## Conclusion

Taken together, the present results may provide further evidence of long-term damage by SARS-CoV-2 to the CNS and more specifically to the limbic system.^3,4,13^ This damage may not be solely explained by ICU effects (as attested by the performances displayed by the moderate group) nor by neuropsychiatric disorders that may arise from the pandemic context and/or personal history of infection such as anxiety, depressive symptoms or PTSD. In the light of the evidence provided in this study, interventions tailored to lessen cognitive impairment induced by SARS-CoV-2 infection could be developed.

## Supplementary Material

fcad177_Supplementary_Data

## Abbreviations

BSR =: bootstrap sampling ratio
FDG =: fluorodeoxyglucose (FDG)
GERT =: Geneva Emotion Recognition Test
ICU =: intensive care unit
PLSC =: partial least squares correlation
PTSD =: post-traumatic stress disorder
