# Supplementary material for: Markers of limbic system damage following SARS-CoV-2 infection
Source: Brain Commun. 2023 Jun 13;5(4):fcad177. doi: 10.1093/braincomms/fcad177 (PMC10320753; doi:10.1093/braincomms/fcad177)
Supplement: fcad177_Supplementary_Data [file fcad177_supplementary_data.docx]

**Supplementary Figure 1. Proportion of multimodal emotion recognition deficits according to symptom severity in the acute phase.**

Emotion recognition performances of a healthy population were considered^1^ and used as a threshold to estimate the proportions of subnormal performances within each group. We then compared the groups’ rates of deficits using chi^2^ tests. Results revealed a significantly higher frequency of subnormal scores (below 5^th^ percentile) for severe patients than for mild ones (*p* < .001), and for moderate patients than for mild ones (*p* = .002), but no difference between moderate and severe patients (*p* = .204), suggesting higher rates of subnormal performances for the severe and moderate groups than for the mild one (see details in SI 2).

**
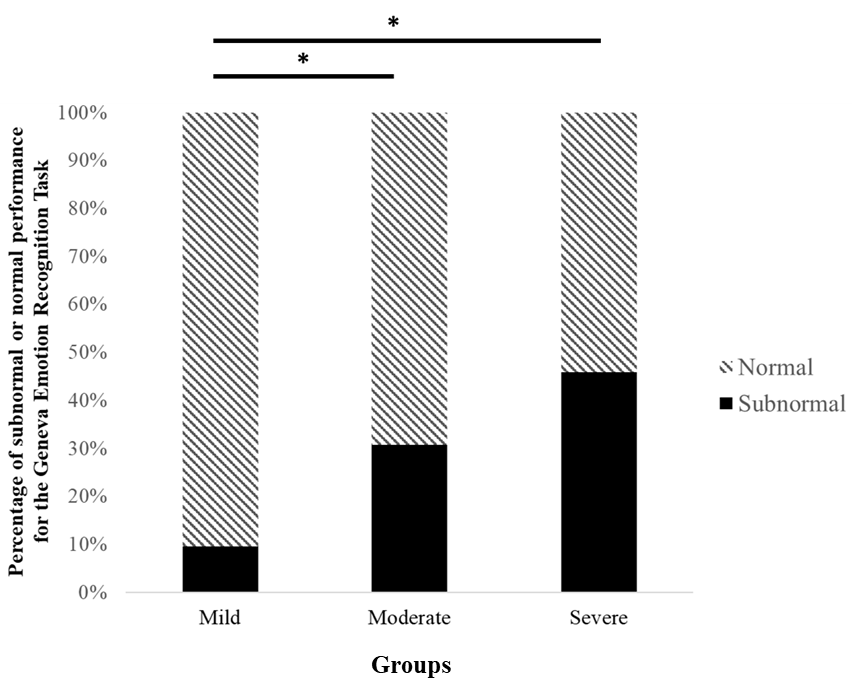
**

- **Significant chi^2^ differences between groups after Bonferroni correction.**

**Supplementary Figure 2. Multivariate latent component 1 from the PLSC analysis without episodic memory scores as a covariate**

**
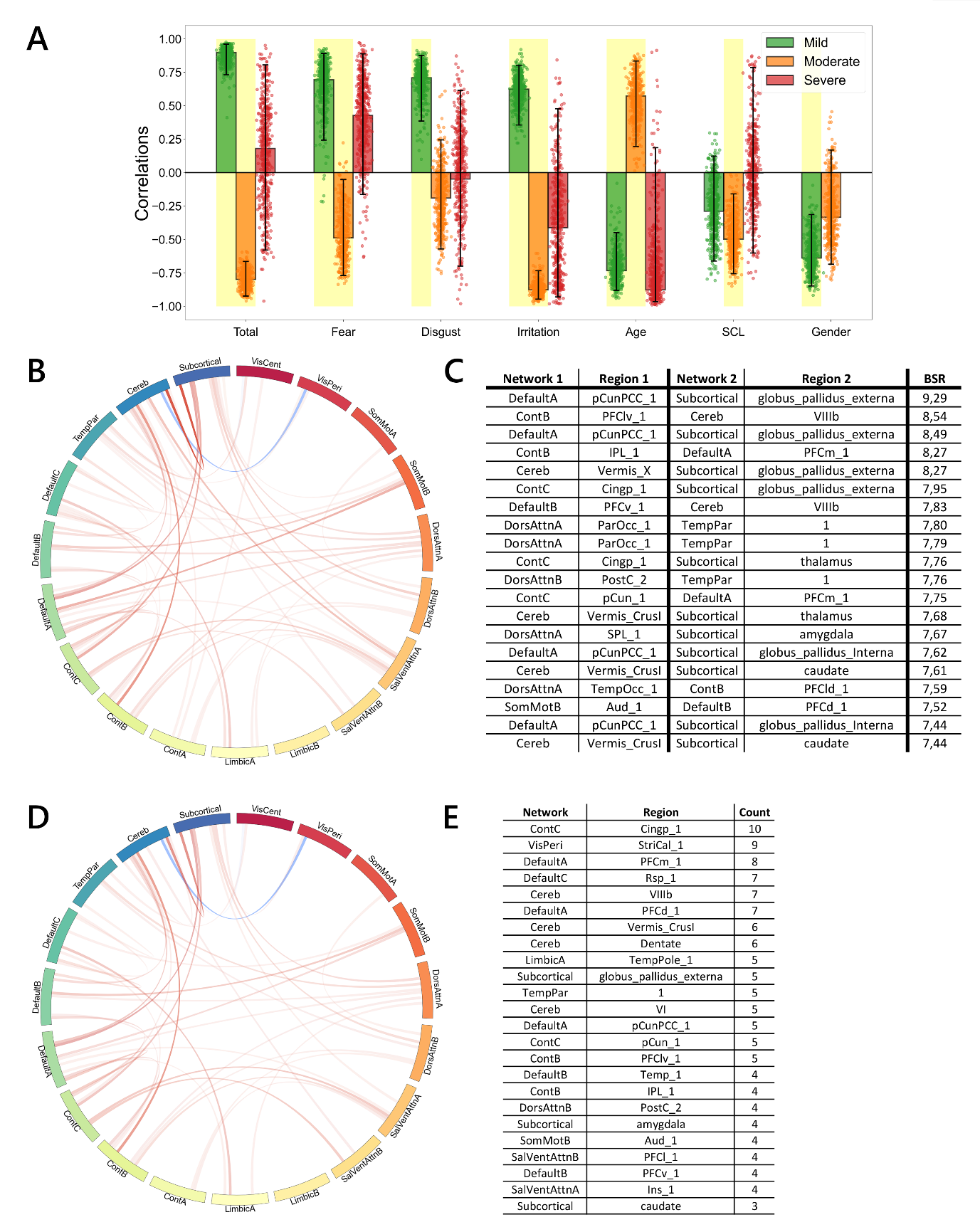
**

The latent component 1 of the group PLSC analyses without episodic memory scores (*p* = 0.004) explained 35.63% of the covariance between behavioural scores and functional connectivity. A) Loadings of behavioural data. Dots represent samples from the bootstrap procedure, and yellow highlights indicate the reliability of the correlations. B) Bootstrap sampling ratios for functional connectivity (5% highest positive and negative values evaluated with bootstrap). This network representation illustrates the neuroimaging pattern, with red links indicating a positive influence of functional connectivity on the latent component, and darker colours indicating a higher number of significant connections involved in the pattern. C) The 20 networks and regions whose connections had the greatest impact (bootstrap sampling ratio [BSR]) on the latent component. D) Network representation of connections overlapping with the functional pattern when episodic memory was included as a covariate. E) List of the 25 regions most closely involved in the overlapping pattern.


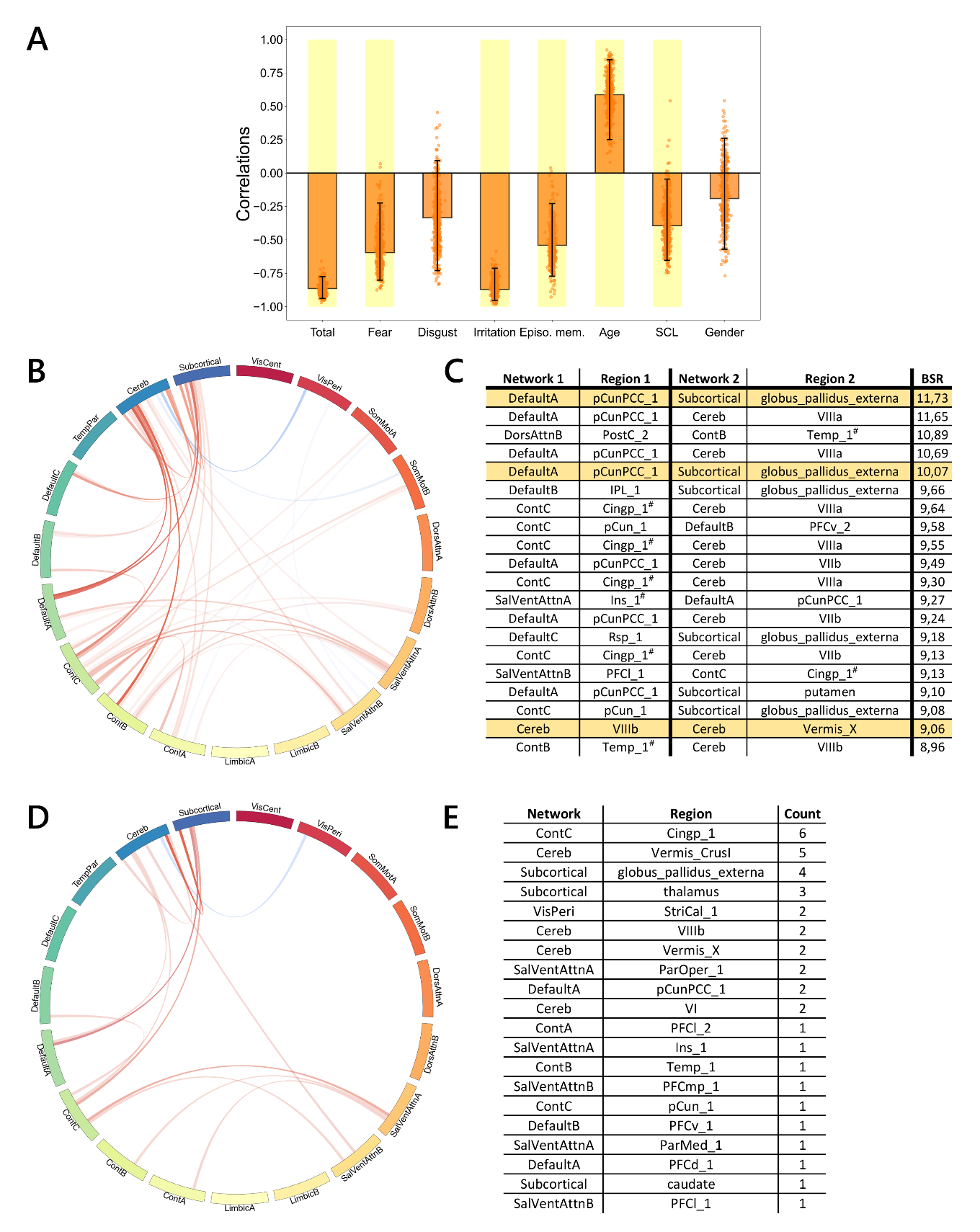
**Supplementary Figure 3.** **Multivariate latent component 1 from the PLSC analysis for moderate patients.**

The latent component 1 of individual PLSC analyses for moderate patients (*p* = 0.021) explained 59.99% of the covariance between behavioural scores and functional connectivity. A) Loadings of behavioural data. Dots represent samples from the bootstrap procedure, and yellow highlights indicate the reliability of the correlations. B) Bootstrap sampling ratios for functional connectivity (5% highest positive and negative values evaluated with bootstrap). This network representation illustrates the neuroimaging pattern, with red links indicating a positive influence of functional connectivity on the latent component, and darker colours indicating a higher number of significant connections involved in the pattern. C) The 20 networks and regions whose connections had the greatest impact (bootstrap sampling ratio [BSR]) on the latent component. D) Network representation of connections overlapping with the group-PLSC functional pattern. E) List of the 25 regions most closely involved in the overlapping pattern.

**Supplementary Information 1.** **MRI preprocessing pipeline**

**Anatomical preprocessing**

Each T1w volume was corrected for intensity nonuniformity using N4BiasFieldCorrection v2.1.0,^2^ and skull-stripped using antsBrainExtraction.sh v2.1.0 (OASIS template). Spatial normalization to the ICBM 152 nonlinear asymmetrical template version 2009c^3^ was performed by means of nonlinear registration, with the antsRegistration tool of ANTs v2.1.0,^4^ using brain-extracted versions of both the T1w volume and the template. Brain tissue segmentation of CSF, white matter, and grey matter was performed on the brain-extracted T1w using FAST^5^ (FSL v5.0.9).

**Functional preprocessing**

Functional data were slice time-corrected using 3dTshift from AFNI v16.2.07,^6^ and motion-corrected using MCFLIRT (FSL v5.0.9^7^). This was followed by FLIRT (FSL) coregistration to the corresponding T1w images using boundary-based registration^8^ with six degrees of freedom. Motion-correcting transformations, BOLD-to-T1w transformation, and T1w-to-template (MNI) warp were concatenated and applied in a single step using antsApplyTransforms (ANTs v2.1.0), configured with Lanczos interpolation. Framewise displacement^9^ was calculated for each functional run using Nipype, and volumes with a framewise displacement greater than 0.7 mm were excluded (SI 3).

Many internal operations of fMRIPrep use nilearn,^10^ principally within the BOLD-processing workflow. For more details of the pipeline, see the section corresponding to workflows in the fMRIPrep documentation.

**Supplementary Table 1.** **Acquisition parameters for structural (T1w MPRAGE) and functional resting state fMRI (rs-fMRI) images.**

T1w MPRAGE:

| Acquisition time | 4 min 36 s |
| --- | --- |
| Repetition time | 2300 ns |
| Echo time | 2.24 ms |
| Acquisition matrix | 256 x 256 |
| FOV | 186 x 239 x 239 mm |
| Flip angle | 8° |
| Number of slices | 208 (sagittal) |
| Voxel size | 0.9 x 0.9375 x 0.9375 mm |
| Slice thickness | 0.9 mm |

*Note.* FOV: field of view.

rs-fMRI:

| Acquisition time | 7 min 59 s |
| --- | --- |
| Repetition time | 1 s |
| Echo time | 30 ms |
| Acquisition matrix | 88 x 88 |
| FOV | 220 x 220 x 160 mm |
| Flip angle | 50° |
| Number of slices | 64 (transversal) |
| Voxel size | 2.5 x 2.5 x 2.5 mm |
| Slice thickness | 2.5 mm |

*Note.* FOV: field of view.

**Supplementary Table 2. Motion and variance of MRI data as a function of severity in the acute phase**

|  | Severity of acute infection | | | |
| --- | --- | --- | --- | --- |
|  | Mild  (*n* = 17) | Moderate  (*n* = 19) | Severe  (*n* = 9) | K-W |
| Mean FD | 0.24 (± 0.12) | 0.25 (± 0.12) | 0.29 (± 0.1) | .379 |
| Excluded volumes | 8.71 (± 20.9) | 13.95 (± 38.65) | 18.11 (± 27.62) | .575 |
| Overall standard deviation of FC | 0.21 (± 0.036) | 0.21 (± 0.029) | 0.21 (± 0.025) | .106 |

*Note*. FD: framewise displacement; FC: functional connectivity; M-W: Mann‑Whitney *U* test; K-W: Kruskal‑Wallis ANOVA.

**Supplementary Table 3. Mean (standard deviation) scores on emotion recognition task variables for each respiratory symptom severity group (mild, moderate, severe). Significant between-group differences on Kruskal–Wallis and Mann–Whitney tests.**

| **Geneva Emotion Recognition Test** | **Mild**  **(mean ± *SD*)** | **Moderate**  **(mean ± *SD*)** | **Severe**  **(mean ± *SD*)** | **Kruskal–Wallis**  ***p* values and Mann-Whitney contrasts** |
| --- | --- | --- | --- | --- |
| Amusement | 0.56 (± 0.28) | 0.60 (± 0.34) | 0.56 (± 0.38) | .813 |
| Anger | 0.74 (± 0.24) | 0.67 (± 0.24) | 0.68 (± 0.23) | .317 |
| Anxiety | 0.58 (± 0.31) | 0.51 (± 0.30) | 0.65 (± 0.23) | .191 |
| Despair | 0.58 (± 0.27) | 0.47 (± 0.32) | 0.49 (± 0.35) | .260 |
| Disgust | 0.61 (± 0.25) | 0.48 (± 0.26) | 0.45 (± 0.26) | **.012*** |
| Fear | 0.37 (± 0.27) | 0.23 (± 0.31) | 0.27 (± 0.30) | **.032^@^** |
| Interest | 0.65 (± 0.34) | 0.54 (± 0.35) | 0.52 (± 0.41) | .281 |
| Irritation | 0.61 (± 0.31) | 0.46 (± 0.34) | 0.30 (± 0.35) | **.002^*^** |
| Joy | 0.67 (± 0.28) | 0.49 (± 0.33) | 0.62 (± 0.38) | .060 |
| Pleasure | 0.67 (± 0.31) | 0.41 (± 0.37) | 0.61 (± 0.36) | .093 |
| Pride | 0.52 (± 0.29) | 0.43 (± 0.31) | 0.35 (± 0.32) | .056 |
| Relief | 0.82 (± 0.24) | 0.80 (± 0.28) | 0.74 (± 0.28) | .520 |
| Sadness | 0.53 (± 0.31) | 0.44 (± 0.27) | 0.45 (± 0.28) | .293 |
| Surprise | 0.58 (± 0.29) | 0.55 (± 0.36) | 0.45 (± 0.34) | .345 |
| Total score | 25.50 (± 5.03) | 21.53 (± 6.79) | 21.48 (± 6.28) | **.004^*@^** |

*Note.* ^*^ Severe patients significantly different from mild patients; ^@^ Moderate patients significantly different from mild patients; ^#^ Severe patients significantly different from moderate patients.

**Supplementary Table 4. Voxel-based morphometry (VBM) results (both for whole brain and for individual brain parcels) according to symptom severity in acute phase. Significance was assessed with analyses of covariance, with age, gender, and sociocultural level as covariates.**

|  |  | **Whole-brain tissue proportions** | | | | | | | | |
| --- | --- | --- | --- | --- | --- | --- | --- | --- | --- | --- |
|  | **Tissue** | **Mild Mean** | **Moderate Mean** | **Severe Mean** | **Whole group, *p*** | **Mild - Moderate, *p*** | | **Mild - Severe, *p*** | | **Moderate - Severe, *p*** |
|  | Grey matter | 0.43333 | 0.42156 | 0.42014 | 0.89726 | 0.59188 | | 0.57276 | | 0.66047 |
|  | White matter | 0.48066 | 0.48205 | 0.48372 | 0.98893 | 0.91686 | | 0.66738 | | 0.89049 |
|  |  |  |  |  |  |  | |  | |  |
|  |  | **Grey-matter voxel proportions** | | | | | | | | |
| **Network** | **Region** | **Mild Mean** | **Moderate Mean** | **Severe Mean** | **Whole group, *p*** | | **Mild - Moderate, *p*** | | **Mild - Severe, *p*** | **Moderate - Severe, *p*** |
| DorsAttnB | PostC_3 | 0.45624 | 0.50858 | 0.45562 | 0.05794 | 0.02928 | | 0.52120 | | 0.19719 |
| DorsAttnB | FEF_1 | 0.52350 | 0.58017 | 0.55253 | 0.08007 | 0.03684 | | 0.11097 | | 0.59040 |
| SalVentAttnA | FrMed_1 | 0.56406 | 0.60312 | 0.58172 | 0.23452 | 0.04896 | | 0.30553 | | 0.66042 |
| SalVentAttnB | PFCmp_1 | 0.69302 | 0.65225 | 0.64314 | 0.21782 | 0.12434 | | 0.02758 | | 0.97649 |
| ContC | pCun_1 | 0.65926 | 0.58687 | 0.59116 | 0.14243 | 0.03492 | | 0.12531 | | 0.71918 |
| SalVentAttnA | FrMed_1 | 0.58103 | 0.62122 | 0.59364 | 0.05957 | 0.04084 | | 0.65534 | | 0.05007 |
| Subcortical | Amygdala | 0.80807 | 0.68738 | 0.73245 | 0.19037 | **0.04562** | | 0.30247 | | 0.25174 |
|  |  |  |  |  |  |  | |  | |  |
|  |  | **White-matter voxel proportions** | | | | | | | | |
| **Network** | **Region** | **Mild Mean** | **Moderate Mean** | **Severe Mean** | **Whole group, *p*** | **Mild - Moderate, *p*** | | **Mild - Severe, *p*** | | **Moderate - Severe, *p*** |
| DorsAttnB | PostC_3 | 0.43885 | 0.35636 | 0.42122 | 0.03840 | 0.01829 | | 0.65796 | | 0.11842 |
| DorsAttnB | FEF_1 | 0.42315 | 0.34245 | 0.37499 | 0.07372 | 0.03151 | | 0.11400 | | 0.41051 |
| ContC | pCun_1 | 0.25471 | 0.33148 | 0.30186 | 0.10005 | 0.03361 | | 0.17863 | | 0.76019 |
| SalVentAttnA | FrMed_1 | 0.33228 | 0.28529 | 0.32855 | 0.06793 | 0.05904 | | 0.94353 | | 0.04602 |
| DefaultA | PFCd_1 | 0.32858 | 0.27826 | 0.34209 | 0.05904 | 0.05657 | | 0.91600 | | 0.04055 |
| Cereb | Vermis_CrusII | 0.35836 | 0.41501 | 0.47450 | 0.51577 | 0.55617 | | 0.04812 | | 0.90971 |
| Cereb | VIIIa | 0.41970 | 0.32971 | 0.40538 | 0.10592 | 0.03131 | | 0.44884 | | 0.72631 |
| Cereb | VIIIb | 0.48916 | 0.36624 | 0.46474 | 0.10518 | 0.04228 | | 0.58133 | | 0.76758 |
| Cereb | IX | 0.43437 | 0.31014 | 0.36195 | 0.02502 | 0.00861 | | 0.09368 | | 0.67954 |
| Cereb | IX | 0.60291 | 0.47490 | 0.53107 | 0.05271 | 0.02515 | | 0.19516 | | 0.93016 |
| Cereb | X | 0.31518 | 0.23113 | 0.29309 | 0.09574 | 0.02426 | | 0.52820 | | 0.25253 |
| Subcortical | Subthalamic_nucleus | 1.00000 | 1.00000 | 1.00000 | 0.99997 | 0.94505 | | 1.00000 | | 0.00399 |

**Regions:** FEF: frontal eye field; FrMed: frontal medial; pCun: precuneus; PFC: prefrontal cortex; PostC: postcentral region.

**References**

1. Schlegel K, Gugelberg HMv, Makowski LM, Gubler DA, Troche SJ. Emotion recognition ability as a predictor of well-being during the COVID-19 pandemic. Social psychological and personality science 2021;12:1380-1391.

2. Tustison NJ, Avants BB, Cook PA, et al. N4ITK: improved N3 bias correction. IEEE transactions on medical imaging 2010;29:1310-1320.

3. Fonov VS, Evans AC, McKinstry RC, Almli CR, Collins D. Unbiased nonlinear average age-appropriate brain templates from birth to adulthood. NeuroImage 2009:S102.

4. Avants BB, Epstein CL, Grossman M, Gee JC. Symmetric diffeomorphic image registration with cross-correlation: evaluating automated labeling of elderly and neurodegenerative brain. Medical image analysis 2008;12:26-41.

5. Zhang Y, Brady JM, Smith S. Hidden Markov random field model for segmentation of brain MR image. Medical Imaging 2000: Image Processing; 2000: International Society for Optics and Photonics: 1126-1137.

6. Cox RW, Hyde JS. Software tools for analysis and visualization of fMRI data. NMR in Biomedicine: An International Journal Devoted to the Development and Application of Magnetic Resonance In Vivo 1997;10:171-178.

7. Jenkinson M, Bannister P, Brady M, Smith S. Improved optimization for the robust and accurate linear registration and motion correction of brain images. Neuroimage 2002;17:825-841.

8. Greve DN, Fischl B. Accurate and robust brain image alignment using boundary-based registration. Neuroimage 2009;48:63-72.

9. Power JD, Mitra A, Laumann TO, Snyder AZ, Schlaggar BL, Petersen SE. Methods to detect, characterize, and remove motion artifact in resting state fMRI. Neuroimage 2014;84:320-341.

10. Abraham A, Pedregosa F, Eickenberg M, et al. Machine learning for neuroimaging with scikit-learn. Frontiers in neuroinformatics 2014:14.
